# Colony Fusion in a Parthenogenetic Ant, *Pristomyrmex* *punctatus*

**DOI:** 10.1673/031.013.3801

**Published:** 2013-04-21

**Authors:** Show Satow, Toshiyuki Satoh, Tadao Hirota

**Affiliations:** 1 Department of Biology, Faculty of Science, Yamagata University, 1-4-12 Kojirakawa-machi, Yamagata-shi, 990-8560, Japan; 2 Department of Veterinary Medicine, Tokyo University of Agriculture and Technology, 3-8-1 Harumi-cho, Fuchushi, Tokyo 183-8538, Japan

**Keywords:** Hymenoptera, Myrmicinae, overwinter, queenless, thelytoky

## Abstract

In the ant *Pristomyrmex punctatus* Smith (Hymenoptera: Formicidae), all young workers lay a small number of eggs parthenogenetically. Some colonies consist of monoclonal individuals that provide high inclusive fitness, according to the kin selection theory. However, in some populations, a majority of the colonies contain multiple lineages. Intracolonial genetic variation of parthenogenetic ants cannot be explained by the multiple mating of single founderesses or by the foundation of a colony by multiple foundresses, which are the usual causes of genetically diverse colonies in social insects. Here, we hypothesized that the fusion of established colonies might facilitate the formation of multiclonal colonies. Colony fusion decreases indirect benefits because of the reduction in intracolonial relatedness. However, when suitable nesting places for overwintering are scarce, colony fusion provides a strategy for the survival of colonies. Here, ants derived from different colonies were allowed to encounter one another in a container with just one nesting place. Initially, high aggression was observed; however, after several days, no aggression was observed and the ants shared the nest. When the fused colonies were allowed to transfer to two alternative nests, ants from different colonies occupied the same nest. This study highlights the importance of limiting the number of nesting places in order to understand the genetic diversity of parthenogenetic ant colonies.

## Introduction

In most animal populations, the vast majority of cooperating individuals are related to each other, which is well explained by the kin selection theory (Hamilton 1964). However, in some avian and mammal species, the cooperators are not related (e.g., kingfisher, Reyer 1984; mongoose, Creel and Waser 1994; manakin, McDonald and Potts 1994; bell miner, McDonald et al. 2008). Furthermore, in social insects, some cooperative societies contain unrelated individuals (Bernasconi and Strassmann 1999). For instance, multiple mating by the queens of harvester ants (*Pogonomyrmex occidentalis*) and honeybees (*Apis mellifera*) results in colonies with low relatedness; however, cooperative societies are maintained (Page 1980; Cole and Wiernasz 1999). In other social insect species, while multiple queens may found a single new colony, the number of queens decreases to one through colony growth. Furthermore, the initial number of queens may influence the survival and development of colonies. For example, in Japanese arboreal ants (*Polyrhachis moesta*), the productivity and survival of offspring are higher in polygynous colonies than in monogynous colonies (Sasaki et al. 2005). In some species, including communal bees (*Lasioglossum hemichalceum* and *Andrena jacobi*), multiple foundresses continue to coexist even after a colony has been established (Kukuk and Sage 1994; Paxton et al. 1996). Such cooperative societies contain unrelated individuals, yet direct mutualistic benefits facilitate their maintenance and growth (Bernasconi and Strassmann 1999; Clutton-Brock 2009).

This type of cooperation among unrelated individuals exists in the parthenogenetic ant *Pristomyrmex punctatus* Smith (Hymenoptera, Formicidae), which reproduces clonally. There are no queens in this ant species. All young females produce a few eggs parthenogenetically and become non-reproductive workers later in life (Mizutani 1980; Itow et al. 1984). Some colonies are monoclonal, and intracolonial relatedness is extremely high (Hasegawa et al. 2001; Nishide et al. 2007). Since large colonies may divide into different colonies, some neighbor colonies are also closely related. However, recent studies using microsatellite markers have shown that some colonies contain multiple genetic strains (Hasegawa et al. 2001; Nishide et al. 2007, 2012; Dobata et al. 2009). For example, in populations from Mie, Japan, the majority of the colonies consist of multiple clones (Dobata et al. 2009).

In social insects, there are four well-known processes by which colonies containing multiple lineages may be established: (1) the queen mates with multiple males, producing workers and reproductives of different genotypes (e.g., honeybees); (2) queens produce their own clones as new queens and their mating partner's clones as drones (e.g., *Wasmannia auropunctata*, Fournier et al. 2005; *Vollenhovia emeryi*, Ohkawara et al. 2006; Kobayashi et al. 2008); (3) unrelated alates (newly mated reproductives) found colonies together (e.g., communal bees; Paxton et al. 1996; Kukuk et al. 2005); and (4) young queens are adopted by or intrude into established colonies (e.g., *Formica* ants, Hölldobler and Wilson 1990, Buschinger 2009; *Leptothorax nylanderi*, Foitzik and Heinze 1998). However, because *P. punctatus* reproduces parthenogenetically, the first and second processes do not occur in this species. With respect to the third process, *P. punctatus* females do not lay enough eggs to establish a new colony alone or in small groups (the average laying rate per worker is 0.14 eggs per day; Tsuji 1994). Furthermore, it takes one year for new workers of *P. punctatus* to lay eggs (Dobata, personal communication). Hence, it is not easy for a colony constructed of a few individuals to survive. The fourth process is also unlikely because young reproductive females usually stay within nests as indoor workers and do not leave nests until becoming non-reproductive outdoor workers (Tsuji 1990); however, indoor workers may also emigrate, but only as part of a large group of nestmates to move nests. Therefore, processes that have been observed in other social insects do not explain the establishment of multiclonal colonies in *P. punctatus*.

In addition, a few “cheater” ants, which are larger and have higher reproductive capacity than “normal” workers, have been documented in some populations of *P. punctatus* colonies (Dobata et al. 2009). Cheater ants are phylogenetically different from normal workers. Therefore, if a large number of cheater ants exist in a colony, genetic research using a few dozen samples would conclude that a colony containing cheater ants is multiclonal. However, the proportion of intracolonial cheater ants was less than 4% in *P. punctatus* (Dobata et al. 2009). In addition, many multiclonal colonies do not contain cheater ants (Nishide, personal communication). Alternatively, if cheater ants produce normal workers, workers of different lineages could coexist in the same colonies. However, Dobata et al. (2009) indicated that cheater ants could not produce phylogenetically different normal ants. Thus, cheater ants might not play important roles in producing multiclonal colonies.

In the current study, the fusion of established colonies was considered as an alternative colony-establishment process (Nishide et al. 2007). For instance, *P. punctatus* is considered a fugitive species (Tsuji 1988a), whereby colonies do not remain at the same nesting place. Instead, nests are temporarily constructed under stones, in decaying logs, or under fallen leaves, with colonies moving from place to place between June and August (Tsuji 1988a). However, fewer nests have been found in these habitats from September to winter (Tsuji 1988a). Like other insects, this ant species utilizes different microhabitats during different seasons (Alonso-Mejía et al. 1992; Barron and Wilson 1998; Shiraishi et al. 2011). For instance, *P. punctatus* overwinters in the deep holes of soils around trees (Tsuji 1988a). This type of habitat is also often occupied by territorial ants (e.g. *Lasius nipponensis, L. japonicas*) throughout the year (Akino et al. 2005; Akino and Yamaoka 2005; Endo and Ichino 2012), although in summer *P. punctatus* nests almost never overlap the territories of other ants. Consequently, *P. punctatus* is exposed to interspecific competition for overwintering places as well as intraspecific competition. Multiple colonies might sometimes encounter one another in limited overwintering places. In this situation, one adaptive strategy is to share nesting places when availability is limited. Although a colony could fight with opponents to monopolize the nest, the cost of fighting is extremely high (Tsuji 1986, 1988b). Field experiments also indicated that a shortage of nesting sites increased queen number per nest in *Solenopsis* ants (McGlynn 2010). Therefore, we hypothesize that colonies of *P. punctatus* are likely to share nests during seasons when the availability of suitable microhabitats is limited, and that such cohabitation might facilitate colony fusion.

The aim of the present study was to investigate how a shortage of nesting places affects hostility among different colonies via three experiments.

### Experiment 1

To evaluate the influence of a shortage of nesting places on hostility, two colonies were allowed to encounter one another in an arena where just one nesting place was available.

### Experiment 2

Even when a shortage of nesting places decreases hostility among colonies, it is possible that such a state is temporary, and high aggressive interactions might be observed after more than one nesting place becomes available. Therefore, two novel nesting places were introduced to the colonies used in Experiment 1, and the behavior of the ants was observed. If the two colonies used different nests and excluded ants of the other colony from their nests, this would indicate that the two colonies did not fuse in Experiment 1. In contrast, if most ants of both colonies aggregated in the same nest, this would indicate that they had become members of a novel unit (colony fusion).

### Experiment 3

The goal was to determine whether a reduction in hostility among colonies in Experiments 1 and 2 was caused by a change in nestmate recognition (cues and/or templates) or by a decrease in aggression against other individuals. Ants were paired in Experiment 2 with (1) ants of the mother colony to which they originally belonged; (2) ants of the mother colony to which the opponents in Experiments 1 and 2 belonged; and (3) ants of other mother colonies. If hostility in (3) was low, this outcome would indicate a reduction in aggression against any individual. If hostility increased in (1) but decreased in (2), this outcome would indicate a change in nestmate recognition.

Through these experiments, it was found that a shortage of nesting places facilitates colony fusion.

## Materials and Methods

### Sample collection and maintenance of mother colonies

**Figure 1.**
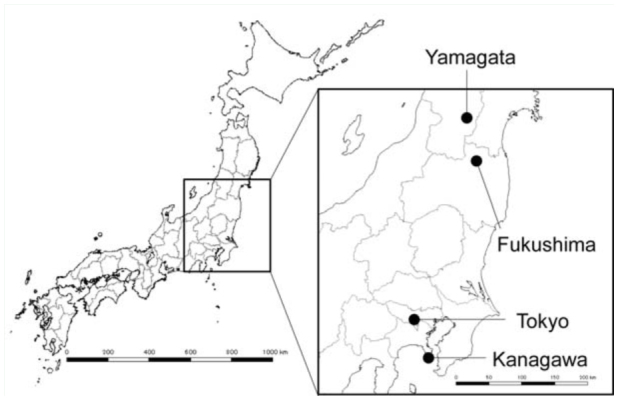
Map of the study area in Japan. Black filled circles indicate the collection sites, which were separated by more than 50 km. High quality figures are available online.

Colonies of *P. punctatus* were collected from under stones, fallen trees, and accumulated fallen leaves at Yamagata, Fukushima, Tokyo, and Kanagawa in Japan from April to August 2008 (Figure 1). Since the collection sites were separated by more than 50 km and *P. punctatus* has no flight ability, there was little possibility that colonies from different sites had recently budded from homologous colonies. Six Yamagata colonies, four Fukushima colonies, two Tokyo colonies, and one Kanagawa colony were used in the experiments. The collected colonies were reared as mother colonies in Fluon™-coated plastic containers (B-15 clear or B-4.5 clear; Iris Ohyama, www.iriseurope.com), the bottoms of which were covered with palm peat (P-8L; Iris Ohyama,). Colonies with more than 10,000 workers were maintained in containers with the dimensions 43.3 × 31.3 × 9.4 cm (B-15 clear). Colonies with less than 10,000 workers were maintained in containers with the dimensions 27.6 × 15.2 × 9.3 cm (B-4.5 clear). One petri dish (diameter: 5 cm, height: 1.5 cm) was placed upside down in the center of each plastic container to serve as a nesting place. The petri dishes were covered with red cellophane, which allowed ants to avoid exposure to light (Burns et al. 2002). Colonies were maintained with a 16:8 L:D photoperiod at 25 ± 1° C. Three grams of sugar and a mixture of honey and yolk were supplied separately every other day.

**Figure 2.**
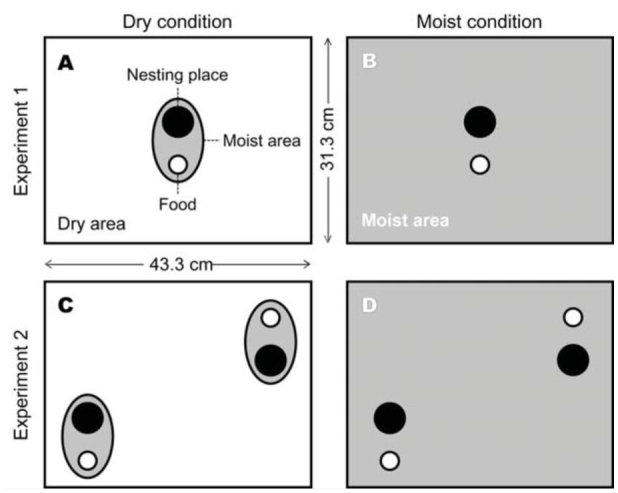
Experimental setup. (A) Dry and (B) moist conditions in Experiment 1. (C) Dry and (D) moist conditions in Experiment 2. Black filled circles and open circles indicate the nesting places and food sites, respectively. Light and dark gray areas represent dry and moist soils, respectively. High quality figures are available online.

Two subgroups of ants were extracted from each mother colony to evaluate how moist conditions around nests influenced hostility. *P. punctatus* prefers moist places rather than dry places. For instance, colonies are usually found in moist places, such as under fallen leaves. Hence, ant behavior might change in response to different soil moisture conditions. Thus, experiments were performed under two types of soil moisture conditions. Experiments were conducted under dry conditions for one subgroup and under moist conditions for the other subgroup. Under dry conditions, only the palm peat under the nesting place and feeding station was kept from drying out, while the rest of the soil was left to dry out naturally (Figure 2A). Under moist conditions, moisture was retained in the palm peat used throughout the container (Figure 2B).

Although *P. punctatus* overwinter in cold conditions, they relocate to overwintering sites when conditions are still temperate (Tsuji 1988a). This activity occurs because ants are ectothermic and cannot actively move under cold conditions. Furthermore, a column of slow-moving ants would be at high risk of predation. Consequently, *P. punctatus* tend to move to overwintering sites, where they encounter different colonies, before air temperatures become low. Hence, the following experiments were conducted at 25 ± 1° C.

Dobata et al. (2009) reported that cheater ants were present in some populations of *P. punctatus* and that these individuals influenced the life-history evolution of this species. Dobata et al. (2009) found that many colonies contained cheater ants within only one specific population. Hence, it was preferred to take into account the presence of cheater ants in the study colonies and their potential influence on the experiments. If cheater ants were present, they would be easily detectable because they have morphological traits that are noticeably different from those of normal workers. However, only three colonies contained a small number of cheater ants, despite collecting 200+ colonies of *P. punctatus* from several dozen populations over a latitudinal range of about 350 km and a longitudinal range of about 450 km in a five-year period. Therefore, an experimental design could not be prepared to include sufficient colonies with cheater ants for statistical analyses.

### Experiment 1: Temporary change of hostility when nesting-place availability was limited

Two colonies collected from different populations were paired to avoid using colonies that shared an immediate ancestor. Four pairs were created from the Fukushima and Yamagata populations, two pairs from the Tokyo and Yamagata populations, one pair from the Kanagawa and Yamagata populations, and one pair from the Fukushima and Tokyo populations. Hostility between the mother colonies of each pair was evaluated before the onset of the experiment. Single ants were selected from two different colonies and transferred to a petri dish. The interaction between opponents was classified into 1 of the following 4 categories: (0) Disregard: No aggressive behavior was observed after the ants recognized each other with their antennae (termed antennation); (1) Avoidance: After antennation, one or both ants moved away from the opponent at faster than walking speed; (2) Threat: One or both ants opened their mandible toward the opponent but did not bite the opponent; (3) Biting: One or both ants bit the opponent.

The most aggressive behavior was recorded within a 5-minute period. Two groups of ten ants were used for each colony pair. The aggression score was 1.93 (± 0.18 SE., n = 80). This result indicates that the colonies paired in the current experiment exhibited high hostility against each other before the experiment.

Two subgroups consisting of 500 ants were removed from each mother colony (dry or moist condition). For each subgroup, 400 ants foraging outside the nest and 100 ants remaining inside the nest were selected from the mother colony. To identify the colony of origin, the dorsal side of the abdomen was painted with a color marker (Mitsubishi paint marker PX-20; orange or white). Painted ants were maintained for a day in a plastic container (15.4 × 11.8 × 11.6 cm), the bottom of which was covered with palm peat. The next day, observations were initiated once two subgroups derived from different colonies were placed in a plastic “experimental container” (43.3 × 31.3 × 9.4 cm), which had a red cellophane-coated petri dish in the center as a nesting place. Across a one-week period, aggression was scored between the subgroups for 1 hr every day at the start of the light-phase photoperiod. Although the behavior of ants was also observed at 4, 7, and 10 hr after the start of the light-phase photoperiod, only the first aggression score of each day was described because aggression scores were consistent within a given day. A white-marked ant and an orange-marked ant walking freely within the container were gently moved to the petri dish, and their behavior was observed for 5 min. An interaction with an opponent was classified into one of the four defined behavioral categories. The most aggressive behavior was recorded within a 5-min period. Ten pairs of ants were observed for each pair of colonies. Ants were returned to the experimental container after observation. Some ants lost their marking, probably due to allogrooming or fighting. These ants were not used in the evaluation of hostility, because the subgroups they belonged to could not be identified.

### Experiment 2: The behavior of ants when two nesting places were available

Although ants derived from different colonies shared a single nesting place in Experiment 1, it was not clear whether their tolerance for one another would be maintained if given the option of additional nest places. If tolerance were temporary, ants would be expected to separate into different colony groups when additional nesting places were provided. Alternatively, ants derived from different colonies might behave as a fused colony. Therefore, after Experiment 1, the petri dish nest was removed from the center and placed two red cellophane-coated petri dishes 15 cm from the center of the container and 30 cm apart (Figures 2C, D). Ants were allowed to move freely for three days, and the number of living ants that remained under each petri dish was counted. A choosiness index (i.e., the proportion of items observed in a given nest) was adopted by using the following formula:

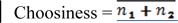

where n1 and n2 are the total number of ants aggregated in Nest 1 and 2, respectively (Stroeymeyt et al. 2010). Choosiness was 1 when all ants aggregated in Nest 1 and 0 when all ants aggregated in Nest 2.

Some ants aggregated between the soil and the wall of the plastic containers rather than under petri dishes. However, very few ants aggregated in this area. Therefore, only ants that aggregated under petri dishes were analyzed.

### Experiment 3: Hostility of fused colonies to mother colonies

One reason for the reduced aggression observed among ants derived from different colonies in Experiments 1 and 2 might be that the experimental conditions caused the ants to become too weak to fight. Therefore, in order to evaluate the aggressive behavior of ants belonging to fused colonies, how they interacted with mother colonies and third-party colonies was observed.

After Experiment 2, the aggressive behavior of ants was evaluated by observing the interactions of two ants in a petri dish. The same observation protocol was used for ant pairs as in Experiment 1; however, the opponent was selected from the following three group-types:

(1) Former nestmates maintained in the mother colony.
(2) Mother colony of an opponent subgroup paired with a target ant in Experiments 1 and 2. For example, when 500 ants of Colony A were paired with 500 ants of Colony B in Experiment 1, the ants in the fused colony that formerly belonged to Colony A were confronted by the ants maintained in Colony B.
(3) A third-party colony of the population that was different from the two colonies paired in Experiments 1 and 2. For 7 of 8 colony pairs, a colony of the Kanagawa population was used as a third party. A colony of the Tokyo population was used as a third-party colony for the pair of colonies collected from the Kanagawa and Yamagata populations.

### Statistical analyses

In Experiment 1, the temporary change in hostility was analyzed using the correlation between several judgments and a criterion ranking, *T_c_*, which ranged from -1 to 1 (Siegel and Casterian 1988). The statistic *T_c_* was equivalent to the average of the Kendall's rank correlation coefficients between each ranker and the common criterion. The correlation between the frequency of fights of each pair and the number of days after the start of the experiments was calculated. *T_c_* was negative when hostility decreased with the number of days in most pairs of colonies, whereas it was positive when hostility increased with the number of days. The influence of moisture conditions on the aggression score was analyzed using a generalized linear mixed model (GLMM) with normal errors. The aggression score was set as a dependent variable. Soil condition (dry or moist) was set as an independent variable. The ID of colony pairs was set as a random factor.

In Experiment 2, choosiness indexes were calculated separately for each subgroup (white or orange-marked subgroups) and analyzed the correlation between the choosiness indexes of two subgroups by using Kendall's tau rank correlation. The correlation coefficient (τ) was positive when both subgroups preferred the same nest. The influence of soil moisture on the number of ants remaining within nesting places and on mortality was first analyzed using GLMM with binomial errors. However, a package ‘glmmML’ (Broström and Holmberg 2011) detected an overdispersion problem. Therefore, those data were also analyzed using Wilcoxon signedrank test. The influence of soil moisture on the proportion was analyzed using GLMM with binomial errors because overdispersion was not detected.

In Experiment 3, the aggression score was analyzed using GLMM with normal errors. The aggression score was set as a dependent variable. The colony to which the opponent belonged (mother colony, opponent mother colony, or third-party colony), the experimental condition (before or after fusion experiment), and the moisture conditions (dry versus moist) were set as independent variables. The ID of colony pairs was set as a random factor. Calculations were performed using the statistical software R 2.15.0 (R Development Core Team 2012).

## Results

### Experiment 1: Temporary change in hostility when nesting-place availability was limited

**Figure 3.**
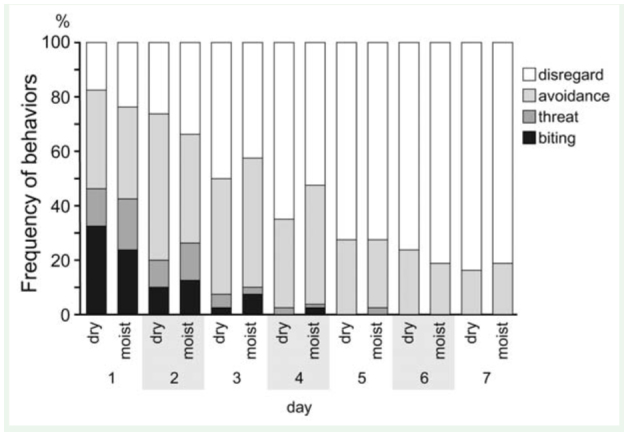
Frequency of behaviors during contact between pairs of ants under dry and moist conditions. Each color bar indicates ant behavior. High quality figures are available online.

Although hostile behavior was frequently observed among ants derived from different colonies just after they were moved into a common container, aggression decreased with the number of days (Figure 3; dry condition, k = 8 colony pairs, n = 7 days, *T_c_* = -0.786, *p* < 0.0001; moist condition, k = 8 colony pairs, n = 7 days, *T_c_* = -0.792, *p* < 0.0001). Although 2.81 ± 0.53 (mean ± SE.) of 10 pairs showed biting behavior on the first day, aggressive behavior reduced with each consecutive day (Figure 3). No aggressive behavior (biting and/or threat) was observed after the fourth day under dry conditions and after the fifth day under moist conditions. Some ants always remained under the red cellophane-coated pepetri dish (i.e., the nesting place), under which ants from different colonies appeared to distribute randomly in all the experiments. Ants from the same colony did not assemble exclusively under the petri dish. In some colony pairs, a small number of ants aggregated between the soil and the wall of the plastic containers, particularly when conditions were moist. These assemblages always contained ants derived from both colonies. Thus, a shortage of nesting places reduced the level of aggression among different colonies and facilitated cohabitation.

The influence of soil moisture on aggression for each colony pair was not significant (GLMM, n = 1120, *p* = 0.398; Figure 3).

### Experiment 2: Ant behavior when two nesting places were available

The ants appeared to be disorientated immediately after the petri dish under which they were inhabiting was removed. However, on the second day, they began to aggregate under the two alternative petri dishes that had been placed at different locations in the container. On the third day, the number of ants in the nesting places was almost the same as the number before the previous nesting place was removed. No aggression was observed.

**Figure 4.**
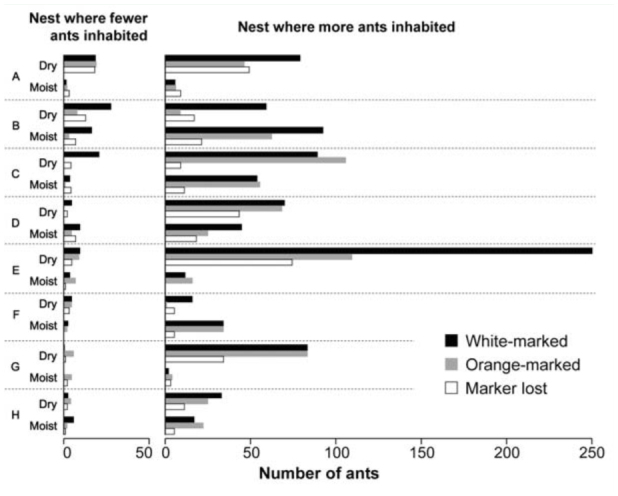
Number of ants that remained in each nest. Black and grey bars indicate white- and orange-marked ants, respectively. Open bar indicates ants for which the marker was lost. Right and left graphs represent nests in which more and fewer ants remained, respectively. High quality figures are available online.

Although ants occupied both nesting places, they predominantly aggregated in one of the two available nesting places (GLMM, z = 19.5, *p* < 0.0001; Figure 4). Subgroups derived from different colonies coexisted in the nesting places occupied by a larger number of ants (Figure 4). Choosiness indexes were positively correlated between the subgroups, regardless of soil moisture conditions (Kendall's tau rank correlation coefficient: for dry conditions, n = 8 colony pairs, τ = 0.764, *p* = 0.008; for moist conditions, n = 8 colony pairs, τ = 0.691, *p* = 0.017). This result indicated that large numbers of ants derived from different colonies chose the same nesting places. Consequently, ants derived from the same colony did not tend to assemble exclusively. Therefore, the cohabitation of ants from different colonies due to the shortage of nesting places was not temporary, since it was maintained after additional nesting places became available.

The total number of ants that remained in the two available nesting places was greater under dry conditions than under moist conditions (Wilcoxon signed-rank test, W = 13, *p* = 0.05). Soil moisture did not influence the probability of colony fusion (GLMM, z = 0.596, *p* = 0.55). From the initiation of Experiment 1 until Experiment 2 completed, 45.5% (S.E. ± 5.28) and 41.2% (SE. ± 5.28) of the ants survived under dry and moist conditions, respectively. Soil moisture did not influence the survival of ants (Wilcoxon signed-rank test, *W* = 29, *p* = 0.62).

### Experiment 3: Hostility of fused colonies to mother colonies

**Figure 5.**
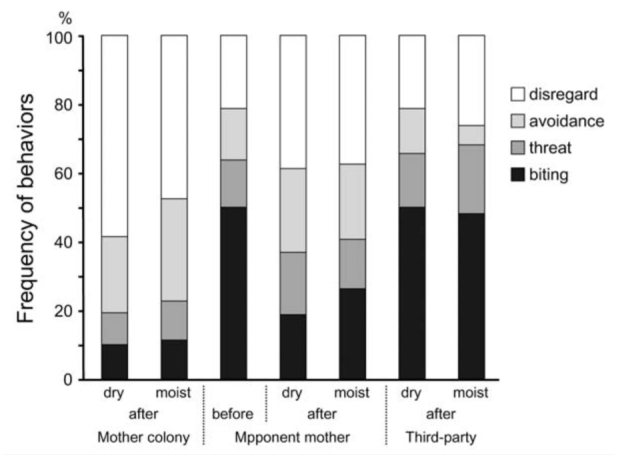
Frequency of behaviors when ants of fused colonies encountered an individual from three types of mother colonies. “Before” was when ants of the mother colony encountered an individual from experimental opponent mother colonies before Experiment 1. “After” was when ants of fused colonies encountered an individual from the three types of mother colonies after the fusion experiment. “Dry” and “moist” indicate soil conditions during the fusion experiment. High quality figures are available online.

When ants encountered an ex-nestmate from their own mother colony after the fusion experiment, aggression scores were 0.68 (SE. ± 0.13) and 0.81 (SE. ± 0.20) under dry and moist conditions, respectively (Figure 5). These aggression scores were significantly higher than 0 (GLMM: for dry, n = 160, *p* < 0.001; for moist, n = 160, *p* < 0.01; Figure 5). Ants of fused or mother colonies initiated an attack. These results indicate that cohabitation with ants derived from different colonies enhanced hostility to former nestmates.

When ants encountered an individual from the mother colony of fusion partners after the fusion experiment, aggression scores were 1.17 (S.E. ± 0.20) and 1.29 (S.E. ± 0.18) under dry and moist conditions, respectively (Figure 5). These aggression scores were significantly lower than the score (1.93 ± 0.18 S.E.) observed between mother colonies of both subgroups before the fusion experiment (GLMM: for dry, n = 240, *p* < 0.0001; for moist, n = 240, *p* = 0.0002; Figure 5). These results indicate that cohabitation in the same nest reduced hostility among partners of the fusion experiment and against the mother colony from which the partners were derived.

When ants encountered an individual from a third-party colony after the fusion experiment, aggression scores were 1.94 (S.E. ± 0.21) and 1.90 (S.E. ± 0.19) under dry and moist conditions, respectively (Figure 5). These aggression scores were not significantly different from the scores (1.93 ± 0.18 S.E.) observed between their own mother colonies and the mother colonies of partners before the fusion experiment (GLMM: for dry, n = 240, *p* = 0.890; for moist, n = 240, *p* = 0.893; Figure 5). Consequently, this result indicates that aggressive behavior was maintained under the conditions of Experiments 1 and 2. Therefore, the observed reduction in aggression was not an artifact of ants becoming weak under artificial conditions.

Soil moisture conditions did not influence aggression scores after the fusion experiment, regardless of opponents (GLMM: for mother colony, *n* = 320, *p* = 0.226; for partner's mother colony, n = 320, *p* = 0.241; for third-party colony, n = 320, *p* = 0.763; Figure 5).

## Discussion

The current study indicated that when suitable nesting places were rare, different colonies aggregated in the same nest. Although intense hostility was observed immediately after ants encountered each other, aggressive interactions decreased with each consecutive day. No biting or threats were observed by the fifth day after the ants initially encountered each other. Ants derived from different colonies were found to coexist and were randomly distributed within the same nest. Previous studies reported that *P. punctatus* strictly discriminate nestmates and show extreme aggression towards other colonies (Tsuji 1986, 1988b). Fighting resulted in the death of a large number of ants, particularly when *P. punctatus* colonies defended their nest from intruders of other colonies in the field (Tsuji 1988b). Sanada-Morimura et al. (2003) demonstrated that *P. punctatus* “remembered” ants from different colonies that they encountered near food resources and showed hostility toward them, even after two weeks. Thus, it has been considered that strong nestmate recognition in *P. punctatus* inhibits individual exchange among different colonies under normal conditions. However, when suitable microhabitats for overwintering are more limited than microhabitats occupied as temporary resting places during summer, ants would be expected to compete for overwintering nests with conspecific colonies. Sharing nests with different colonies might be the second best strategy when it is difficult to locate empty microhabitats, because the cost of fighting to eliminate different colonies from these microhabitats is too high. Thus, the results of the current study are consistent with the hypothesis that overwintering conditions facilitate colony fusion in *P. punctatus*.

In Experiment 2, the coexistence observed in Experiment 1 was tested to determine whether it was temporary. If the coexistence of individuals derived from different colonies was temporary, ants would be expected to separate into two groups, sharing a natal colony when two empty nesting places are available to them. However, individuals from different colonies moved together and coexisted in the same nest. In most pairs of colonies, the majority of the ants were observed to aggregate in one of the two nests. This indicates that most ants from different colonies were fused into one cohesive group. Ants were not forced but were allowed to share the nest voluntarily when nesting places were limited. This result indicates that *P. punctatus* colonies fuse more frequently during the pre-wintering period than expected.

In Experiment 3, hostility to third-party colonies did not change before or after colony fusion. This result indicated that the fusion experiment did not reduce the general aggression of ants. Hostility was reduced against the mother colony from which the fusion partners were derived. In addition, ants of fused colonies showed aggression against their own former nestmates. Ants of both fused colonies and mother colonies initiated an attack. These results indicate that fusion experiments influence both cues and templates underlying nestmate recognition. For instance, ants of mother colonies attacked ex-nestmates in fused colonies, probably because contact with fusion partners changed the cues (e.g., cuticular hydrocarbon profiles) of the ex-nestmates in the fused colonies. Ants of fused colonies attacked ex-nestmates in mother colonies probably because contact with fusion partners changed the templates of the ants in the fused colonies. Thus, nestmate recognition of *P. punctatus* might depend on environmental factors, as reported in many ant species, although genetic factors might also be involved (ElShowk et al. 2010).

In the present study, high mortality of ants was observed (approximately 55% of ants). Ants died mainly due to intercolonial battles a few days after both colonies encountered each other. High mortality of ants caused by battles was also observed in the field (Tsuji 1988b). High mortality of nestmates might be one of the factors facilitating neutral or peaceful interactions between surviving ants. However, surviving ants exhibited high hostility to thirdparty colonies in Experiment 3. Consequently, ants would lose hostility specifically to partners of fusion experiment.

In the termite *Nasutitermes corniger*, polygamous colonies are less aggressive toward foreign colonies than monogamous colonies, probably because high intracolonial diversity increases tolerance to various signals (Adams et al. 2007). If intracolonial diversity also influenced the aggression of *P. punctatus*, fused colonies would be less aggressive than mother colonies before fused experiments. However, Experiment 3 showed that aggression against foreign colonies did not change before or after the fusion experiments. Further experiments are necessary to understand the influences of intracolonial diversity on the aggression of *P. punctatus*.

A habituation effect might also explain why two different colonies decreased hostility towards each other on each consecutive day of Experiment 1, whereby repeated encounters with non-nestmates reduced ant response to different odors. However, even if ants become habituated to non-nestmates, their response to ex-nestmates should not change. Consequently, a habituation effect could not explain why ants of fused colonies became aggressive toward ex-nestmates of mother colonies. Therefore, a habituation effect might not be the predominant cause of colony fusion.

Colony fusion was also observed in the Argentine ant *Linepithema humile* under laboratory conditions (Vásquez and Silverman 2008). However, some colony pairs showed intense hostility throughout the experiments and did not fuse. Colony fusion was only observed in pairs that showed fewer aggressive interactions immediately after encounter. Conversely, in the present study, aggression was high in all colony pairs just after encounter, but then it gradually reduced. Colony fusion was observed in most of the study pairs. One reason why colony fusion was observed more frequently in *P. punctatus* than in *L. humile* is a difference in experimental design. In the present study, limited nesting places were supplised to the ants, and whether or not they shared nests was evaluated. In contrast, Vásquez and Silverman (2008) maintained each colony of *L. humile* in a separate container with a nesting place and examined whether two colonies gathered into one of the containers after the two containers were connected with a hose. If only one suitable nesting place was available to the two colonies, *L. humile* might have shown similar dynamics in hostility and a higher frequency of colony fusion, as observed in the present study.

In *L. humile*, amicable fusion was observed between genetically related colonies (Vásquez and Silverman 2008) because related colonies had similar cuticular hydrocarbon profiles (Vásquez et al. 2009). In *Temnothorax* ants, colony fusion occurs in species with low intercolonial genetic diversity, whereas colony fusion is uncommon in species that have high intercolonial genetic diversity (Foitzik et al. 2007; Franks et al. 2007). However, in the fusion experiment, colonies collected from different populations separated by more than 50 km were paired. Since *P. punctatus* does not have a nuptial flight, gene flow is expected to be extremely low among different populations in the wild. Consequently, it is unlikely that two colonies that were genetically related in the fusion experiments were paird. In addition, *P. punctatus* often fight intensely against colonies that share genetic traits (Yokoyama, unpublished). Therefore, the results of the current fusion experiments had a high probability of being independent of genetic similarity among colonies.

In the African army ant, *Dorylus molestus*, colonies frequently fused with neighbor colonies after queen loss (Kronauer et al. 2010). Although *P. punctatus* does not have queens, different colonies show high hostility to each other (Tsuji 1988b). Therefore, queen loss does not explain why *P. punctatus* colonies fused.

Kellner et al. (2010) reported colony fusion between colonies that established nests in the parthenogenetic ant *Platythyrea punctata*. However, *Pl. punctata* behavior in the process of colony fusion was different from the ant used in the present study, *P. punctatus. Pl. punctata* colonies fused into a unit apparently peacefully, but after colony fusion, reproductives of only one linage could survive and other reproductives were killed. This was not the case in *P. punctatus*, which fought between different colonies immediately after they encountered each other. However, reproductive young ants in *P. punctatus* aggregated tolerantly together regardless of lineages. Comparison of life history traits between *P. punctatus* and *Pl. punctata* would reveal what kinds of ecological factors shaped social interactions in the process of colony fusion.

High intracolonial genetic diversity increases the fitness of colonies in some social insects. In polyandrous harvest ants, within-colony relatedness is correlated negatively with colony growth rate (Cole and Wiernasz 1999). In the honeybees, colonies containing multiple patrilines have higher productivity and survival than colonies with single patrilines (Mattila and Seeley 2007). When the most suitable genotypes are different for types of tasks, genetic diversity would have positive influences on colonies exposed to fluctuating environments (Jones et al. 2004). In *Pl. punctata*, colony fusion would result in benefits for the genetic lineage whose reproductives survive (Kellner et al. 2010). Increased heterogeneity would be beneficial in terms of parasite resistance, productivity, task efficiency, and division of labor (Liersch and Schmid-Hempel 1998; Jones et al. 2004; Oldroyd and Fewell 2007; Smith et al. 2008). Therefore, an increase of intracolonial diversity by colony fusion might also influence the fitness of colonies in *P. punctatus*.

Some researchers have suggested that a higher temperature (> 28° C) is necessary to induce *P. punctatus* workers to behave aggressively under laboratory conditions (Tsuji and Sanada-Morimura, personal communications). However, in the present experiments, intense aggression was observed between fused colonies and third-party colonies, and these experiments were performed in a room maintained at 25° C. Therefore, this temperature was not low enough to inhibit aggression. In addition, strong hostility of fused colonies towards foreign colonies indicates that the possibility of aggressive individuals being killed at the initial stages of fusion experiments must be rejected.

In the present experiment, *P. punctatus* exhibited high hostility to other colonies and yet were observed to fuse with these colonies when the availability of suitable nesting places was scarce. This observation is consistent with field research in which multiple strains within colonies were detected despite *P. punctatus* reproducing parthenogenetically. When suitable nesting places are scarce, the suppression of aggression might be adaptive to secure rare nests and avoid costly fights during the overwintering period. Therefore, colony fusion might evolve if the benefit of overwintering success exceeds the indirect cost of allowing ants of different strains to reproduce within their colonies. Further studies are necessary to understand the direct influences of colony fusion on fitness.
